# Supplementary material for: Structure shows that the BIR2 domain of E3 ligase XIAP binds across the RIPK2 kinase dimer interface
Source: Life Sci Alliance. 2023 Sep 6;6(11):e202201784. doi: 10.26508/lsa.202201784 (PMC10485824; doi:10.26508/lsa.202201784)

Source data for Figure 4

Source data for panel 4B

Total (Blot: HA, MYC and β-actin)

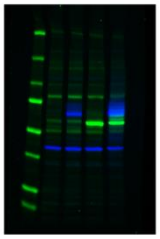

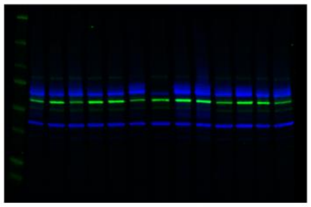

HA-IP (Blot: HA, MYC)

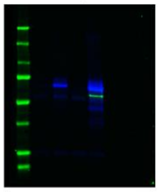

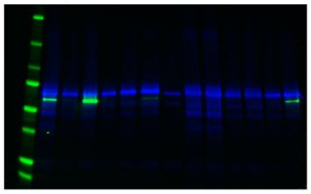

Source data for panel 4C

Total (Blot: HA, MYC and β-actin)

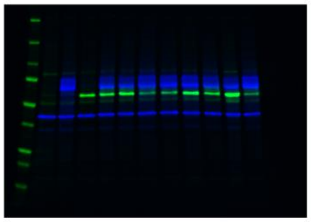

MYC-IP (Blot: HA, MYC)

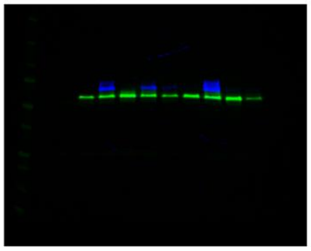

Source data for panel 4D

Blot RIPK2

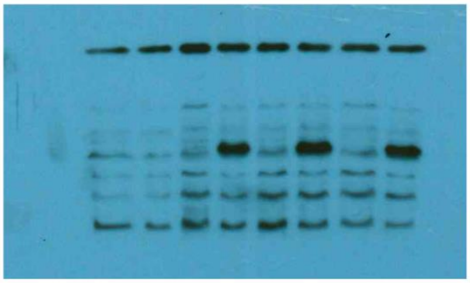

Blot GAPDH

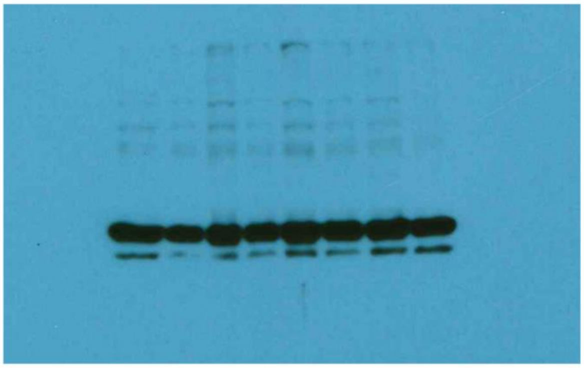

Supplement: Supplementary file 3 [file LSA-2022-01784_SdataF4.pdf]
