# Supplementary material for: Structure shows that the BIR2 domain of E3 ligase XIAP binds across the RIPK2 kinase dimer interface
Source: Life Sci Alliance. 2023 Sep 6;6(11):e202201784. doi: 10.26508/lsa.202201784 (PMC10485824; doi:10.26508/lsa.202201784)

Source data for Figure S9

RIPK2$^{1\text{-}317}$-XIAP BIR2$^{154\text{-}240}$

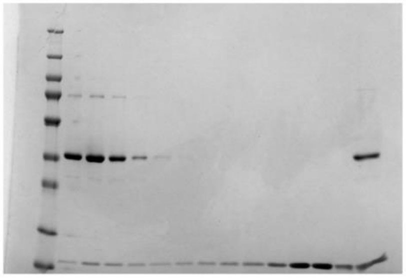

RIPK2$^{1\text{-}317}$ K209A-XIAP BIR2$^{154\text{-}240}$

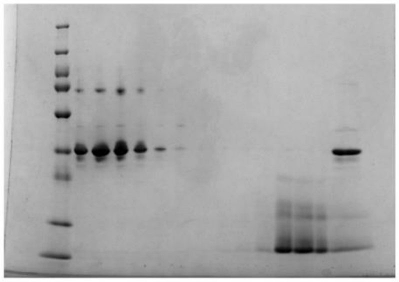

RIPK2$^{1\text{-}317}$ K209R-XIAP BIR2$^{154\text{-}240}$

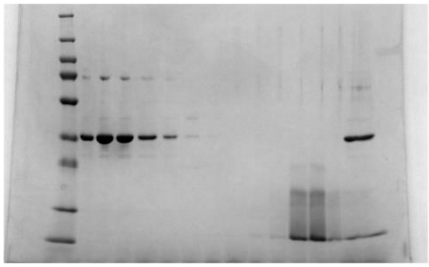

RIPK2$^{1\text{-}317}$ S282L-XIAP BIR2$^{154\text{-}240}$

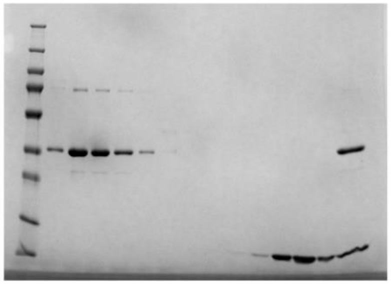

Supplement: Supplementary file 4 [file LSA-2022-01784_SdataFS9.pdf]
